# Cytochrome P450 2D6 profiles and their relationship with outcomes of primaquine anti-relapse therapy in Australian Defence Force personnel deployed to Papua New Guinea and East Timor

**DOI:** 10.1186/s12936-019-2774-2

**Published:** 2019-04-18

**Authors:** Nanhua Chen, Simone Dowd, Michelle L. Gatton, Alyson Auliff, Michael D. Edstein, Qin Cheng

**Affiliations:** 1 Australian Defence Force Malaria and Infectious Disease Institute, Brisbane, QLD Australia; 2 School of Public Health and Social Work, Queensland University of Technology, Brisbane, QLD Australia

**Keywords:** *Plasmodium vivax*, Relapses, Primaquine, CYP2D6 allele, CYP2D6 phenotype, CYP2D6 activity score

## Abstract

**Background:**

Primaquine, an 8-aminoquinoline with anti-hypnozoite activity against *Plasmodium vivax*, is metabolized by human cytochrome P450 2D6 (CYP2D6) to its active metabolite. Human CYP2D6 activities may influence the metabolism of primaquine and the risk of experiencing *Plasmodium* relapses following primaquine anti-relapse therapies (PART). In this study, the CYP2D6 profile and its relationship with outcomes of PART in Australian Defence Force (ADF) personnel is retrospectively investigated.

**Methods:**

Genomic DNA was isolated from stored and de-identified serum or blood samples from ADF personnel deployed on peacekeeping duties to Papua New Guinea (PNG) (1999) and East Timor (1999–2000) who received PART before returning to Australia and after experiencing relapses. CYP2D6 allelic type was determined by PCR and Sanger sequencing. CYP2D6 allele frequency, predicted phenotypes and activity scores were compared among personnel who did not experience *P. vivax* (ADF-NR, n = 48) and those who experience at least one (ADF-R, n = 109) relapse, as well as between those who experienced 1 (n = 79), 2 (n = 21) and 3–5 (n = 9) relapses within the ADF-R group.

**Results:**

16 CYP2D6 alleles were observed in 157 ADF personnel. Alleles *1, *4, *2 and *41 were major alleles (> 5%). The CYP2D6 allele frequency profile in the ADF-NR group matched that of a European population. There was an increased proportion of non-functional CYP2D6 alleles in the ADF-R group compared to the European population and ADF-NR group. However, frequencies of predicted CYP2D6 phenotype and activity score were not different between the ADF-R and ADF-NR groups, nor among sub-groups experiencing multiple relapses within the ADF-R group.

**Conclusions:**

CYP2D6 phenotype or activity score based on the allele classification was not a major contributor to *P. vivax* relapse in this ADF cohort. Other factors such as adherence and/or parasite tolerance to primaquine are likely contributing factors to *P. vivax* relapses in this cohort.

## Background

*Plasmodium vivax* infection is a major cause of human malaria in areas outside Africa [1, 2], and presents a major challenge to the global malaria elimination programmes due to several underlying biological characteristics of this parasite species. Compared to *Plasmodium falciparum* infections, patients with *P. vivax* often have lower parasitaemias leading to a higher proportion of cases below the limit of detection for microscopy and rapid diagnostic tests (RDTs). There is also a higher proportion of asymptomatic and sub-microscopic carriers in communities who contribute to transmission [3]. *Plasmodium vivax* parasites produce gametocytes before the appearance of clinical symptoms, and throughout the course of infection leading to more effective transmission. Importantly, a single *P. vivax* infection can cause many episodes of relapses days and months after the original infection due to activation of hypnozoites deposited in the liver of the infected host at the time of the original infection. It has been shown that relapses contribute to ~ 80% of all *P. vivax* infections in Papua New Guinea (PNG) [4]. As a result, *P. vivax* is more resilient to malaria control measures and countries have reported increases in proportion of *P. vivax* infections as they progress from malaria control to elimination [5–7].

*Plasmodium vivax* relapses can be prevented by using drugs that are capable of killing hypnozoites in the liver. Until recently the only licenced drug for this purpose has been primaquine, an 8 aminoquinoline. The World Health Organization (WHO) recommends that *P. vivax* infected patients (except pregnant or breast feeding women, infants and glucose-6-phosphate dehydrogenase (G6PD) deficient individuals) should receive a 14 day course of primaquine radical cure (0.25–0.5 mg/kg body weight daily) to prevent relapses [8]. However, wide use of primaquine is associated with several major barriers. Firstly, primaquine can cause haemolysis in people with deficient G6PD activity. Therefore, primaquine use requires quality point of care G6PD tests. Secondly, the recommended primaquine regimen for preventing relapses is daily primaquine for 14 days and as such often suffers from poor adherence [9–12]. Thirdly, while there is no confirmed evidence of primaquine resistance, it has been documented that *P. vivax* strains in areas of Oceania are tolerant to primaquine [13] with higher doses of primaquine required to effectively prevent relapses [14–16]. Finally, recent publications reported that human cytochrome P450 2D6 (CYP2D6) enzyme is essential for the metabolism of primaquine into its active metabolites [17, 18], suggesting that reduced primaquine metabolism may be associated with increased risk of *P. vivax* relapses.

The CYP2D6 activity phenotype can be measured by challenging individuals with a probe substrate, and results are categorized as ultrarapid metabolizer (UM), extensive metabolizer (EM), intermediate metabolizer (IM), and poor metabolizer (PM) [19]. CYP2D6 phenotype can also be predicted by determining the allelic type of the CYP2D6 coding gene. The CYP2D6 gene is polymorphic with over 150 allelic variants defined [20] (https://www.pharmvar.org/gene/CYP2D6). These variants are classified as increased function (IF), fully function (FF), reduced function (RF) and non-function (NF) alleles. However, prediction of CYP2D6 phenotypes from CYP2D6 allelic types can be challenging due to the number of allelic variants and the resulting complexity of combinations. An activity score (AS) system was developed, in that an IF, FF, RF and NF allele receives a score of 2, 1, 0.5 and 0, respectively [20]. This helps to simplify the allelic type interpretation.

There is a considerable variation in the frequency of CYP2D6 alleles among different ethnic groups [19, 21, 22], with variations that translates into CYP2D6 phenotypes. For instance, the proportions of CYP2D6 IM and PM phenotypes are estimated to be 10–15% and 5–10%, respectively, for Caucasians [23, 24]. In Asians, the CYP2D6 PM phenotype is rare, however, the IM phenotype is about 50% [22].

Several studies have demonstrated an association between CYP2D6 phenotypes and risk of *P. vivax* relapse. A human experimental challenge trial reported that volunteers with PM and IM CYP2D6 phenotypes had an increased risk of suffering from relapses [25]. A traveller experiencing multiple *P. vivax* relapses despite receiving primaquine radical cure at each episode was found to have a CYP2D6 IM phenotype [26]. Field studies conducted in Brazil [27, 28] and Indonesia [29] suggested that the PM and IM phenotypes or AS below 1.5 and 1, respectively, are associated with increased risk of recurrence of vivax malaria. In contrast, a case study failed to identify mutations at four known mutation sites within CYP2D6 in a patient who failed multiple times after receiving 30 mg of primaquine daily for 14 or 28 days, suggesting primaquine resistance [30].

*Plasmodium vivax* is also a major health problem for military personnel deployed to malaria endemic areas. While prophylaxes regimens offer effective malaria prevention for deployed troops in endemic countries, primaquine anti-relapse therapy (PART) have not been fully effective in preventing relapses. Relapses of *P. vivax* malaria in military personnel after returning home are common. In Australian Defence Force (ADF) personnel deployed to East Timor, over 400 cases of relapsing vivax malaria were documented in returned troops despite receiving PART [15]. A number of relapsing *P. vivax* infections were also documented in personnel returning from Bougainville, PNG [31]. A subgroup of personnel experienced more than one relapses despite receiving PART after the first relapse. The reasons underlying failures of PART in the ADF cohort are not fully understood.

To understand whether CYP2D6 activity was an identifiable factor in this cohort of ADF personnel who failed PART with *P. vivax* relapses after returning to Australia following deployment to East Timor and PNG, the CYP2D6 allelic profile and relationship between CYP2D6 allele and *P. vivax* relapses was retrospectively investigated in the ADF cohort. Predicted CYP2D6 phenotype and activity scores were compared between the ADF personnel who experienced *P. vivax* relapses and those who did not, and within ADF personnel experiencing different number of relapses. It is anticipated that these findings will help to improve the effectiveness of PART and reduce the risk of *P. vivax* relapses in returning troops.

## Methods

### Study cohort and samples

Stored, de-identified serum or blood samples from 157 ADF personnel deployed on peacekeeping duties to Bougainville, PNG in 1999 and East Timor in 1999–2000 were included in this retrospective study. All personnel who were G6PD normal (determined at enlistment and recorded in personal medical records) received unobserved presumptive PART consisting of doxycycline (100 mg daily for 14 days) and primaquine (7.5 mg 3 times daily or 15 mg twice daily for 14 days, without body weight adjustment) commencing before their return to Australia. The sample set was specifically selected based on whether personnel experienced vivax malaria following their return to Australia. A set of serum samples was selected by computer generated randomization from stored samples from personnel who did not experience *P. vivax* (non-relapse, ADF-NR group, n = 48) after returning to Australia. The second set comprised samples from all ADF personnel who experienced at least one episode of *P. vivax* after returning to Australia and had a serum or blood sample (ADF-R group, n = 109). Within the ADF-R group, 79, 21 and 9 individuals experienced 1, 2 and 3–5 relapses, respectively. PART at each relapse included primaquine (7.5 mg 3 times daily or 15 mg twice daily for 14 days) for radical cure.

Serum samples that were collected in PNG before PART were stored in liquid nitrogen and transferred to the Australian Defence Force Malaria and Infectious Disease Institute (ADFMIDI) where the samples were stored at − 80 °C. Blood samples were collected, transported to ADFMIDI and stored at − 80 °C.

*Plasmodium vivax* infections were diagnosed by microscopy at the time of malaria onset. A subset of samples from East Timor were confirmed as malaria-positive by PCR and molecular genotyping [32].

### DNA isolation and sequencing

Genomic DNA was isolated from human serum or blood preserved in 6 M guanidine-HCL (1:10) using a QIAamp DNA Mini Kit according to manufacturer’s instructions (QIAGEN). Full length CYP2D6 was amplified with nested PCR using PfuUltra II Fusion HS DNA Polymerase (Aligent) (Table 1). PCR product was then treated with ExoSAP-It (Affymetrix). To determine the SNPs that define specific allelic types of CYP2D6 (https://www.pharmvar.org/gene/CYP2D6), 7 primers (Table 1) were used to carry out Sanger sequencing using Big Dye v3.1 (Applied Biosystems). Deletions and amplifications of the 2D6 gene were detected following published protocols [33].

**Table 1:** Primers used for PCR and sequencing *CYP2D6*

Purpose	Primer (position, bp)	SNPs/indels to detect	Primer sequence (5′–3′)	PCR condition	Ref
Nested PCR Round 1	2D6-DPKup (− 352 to − 320)	Full length	GTT ATC CCA GAA GGC TTT GCA GGC TTC A	95 °C 1 min; 95 °C 20 s, 65 °C 20 s, 72 °C 2 min 30 s, 50 cycles	[34]
	2D6-DPKlow (4763 to 4743)	Full length	GCC GAC TGA GCC CTG GGA GGT AGG TA		
Nested PCR Round 2	2D6gene sense (− 270 to − 239)	Full length	GGC GGC CTA CCC TGG GTA AGG GCC TGG AGC AGG A	95 °C 1 min; 95 °C 20 s, 72 °C 2 min 30 s, 40 cycles	[35]
	2D6gene antisense (4413 to 4382)	Full length	CTC AGC CTC AAC GTA CCC CTG TCT CAA ATG CG		
Sanger sequencing	2D6Ex1FH (− 126 to − 103)	31G>A, 77G>A, 100C>T, 124G>A, 137_138insT	CAG CTC CCT TTA TAA GGG AAG GGT	96 °C 2 min; 96 °C 10 s, 50 °C 5 s, 60 °C 4 min, 30 cycles	[34]
	2D6-SF4 (695 to 717)	883G>C, 1023C>T, 1039C>T	CCA AAC TGA GTT CCT CCA TCA CA		
	2D6-SF6 (1417 to 1436)	1584delG, 1659G>A, 1661G>C, 1707delT, 1716G>A, 1758G>T	AGA GAC GAG GTG GGG CAA AG		
	2D6Ex4F1s (1780 to 1797)	1846G>A, 1863_1864insTTTCGCCC, 1973_1974insG, 1978C>T, 1979T>C, 2291G>A	ACA AAG CGG GAA CTG GGA		
	2D6Ex5F2s (2318 to 2339)	2539_2542delAACT, 2549delA, 2573_2574insC, 2587_2590delGACT, 2615_2617delAAG	TTG GTG AGG TCA GTG GTA AGG A		
	2D6-SF11 (2735 to 2756)	2850C>T, 2935A>C, 2950G>C, 2988G>A, 3183G>A, 3201C>T, 3259_3260insGT	TGA CAG GTG CAG AAT TGG AGG T		
	2D6-Ex9F2s (3920 to 3938)	4125_4133insGTGCCCACT, 4180G>C	CCT TCC TGC CTT TCT CAG C		
2D6 duplication detection	2D6dup sense	Gene duplication	CCT GGG AAG GCC CCA TGG AAG	95 °C 2 min; 95 °C 20 s, 68 °C 20 s, 72 °C 2 min, 40 cycles	[33]
	2D6dup antisense	Gene duplication	CAG TTA CGG CAG TGG TCA GCT		
2D6 deletion detection Round 1	2D6del01 sense	Gene deletion	GTT GGA GCT CCT GAC CTC TTC	95 °C 2 min; 95 °C 20 s, 60 °C 20 s, 72 °C 2 min, 50 cycles	This study
	2D6del antisense	Gene deletion	TAT ATG CCA GGG CTA CCT CCC		
2D6 deletion detection Round 2	2D6del sense	Gene deletion	ACC GGG CAC CTG TAC TCC TCA	95 °C 2 min; 95 °C 20 s, 68 °C 20 s, 72 °C 2 min, 40 cycles	[33]
	2D6del antisense	Gene deletion	GCA TGA GCT AAG GCA CCC AGA C		

### CYP2D6 allele determination

Sequence polymorphisms and gene copy number variations were identified allowing two allelic types of CYP2D6 to be determined for each sample. Homogenous and heterogeneous sequences at each polymorphic loci were recorded and their allelic types were defined following the CYP2D6 naming convention outlined by the Pharmacogene Variation (PharmVar) Consortium (https://www.pharmvar.org/gene/CYP2D6).

### CYP2D6 phenotype prediction

Phenotype prediction from CYP2D6 alleles were made following the matrix used in [26]. Briefly, individuals having at least one increased function (IF) allele or one FF allele regardless of the function of the second allele were predicted as UMs and EMs respectively, while those with two NF alleles were predicted as PMs. Individuals having two RF alleles or one RF plus one NF allele were predicted as IMs.

### CYP2D6 activity score (AS) determination

An AS is assigned to each CYP2D6 allele detected following Gaedigk’s method [20, 33]: 2 for an IF allele, 1 for a FF allele, 0.5 for a RF allele and 0 for a NF allele. AS for an individual sample was the sum of AS of the two CYP2D6 alleles detected in that sample.

### Statistical analyses

Comparisons of allele frequency were conducted using Chi-square Goodness of Fit test. A generalized linear model with a log link function was used to assess whether phenotype was a significant predictor of relapse. A receiver operating characteristic curve (ROC) analysis was conducted to determine the optimal CYP2D6 AS threshold to separate ADF-R from ADF-NR. Fishers exact test was used to test for an association between AS and ADF group, while Chi-squire test (Linear-by-Linear Association) was also used to compare AS distribution between ADF-R sub-groups with 1 relapse to sub-group with ≥ 2 relapses. These analyses were performed using GraphPad Prism (version 6.05) and SPSS (V25).

### Human ethics

The retrospective analysis of archived and de-identified samples was approved by Australian Defence Human Research Ethics Committee (ADHREC 288-02 and 802-15).

## Results

### CYP2D6 alleles and allele frequency

**Fig. 1:**
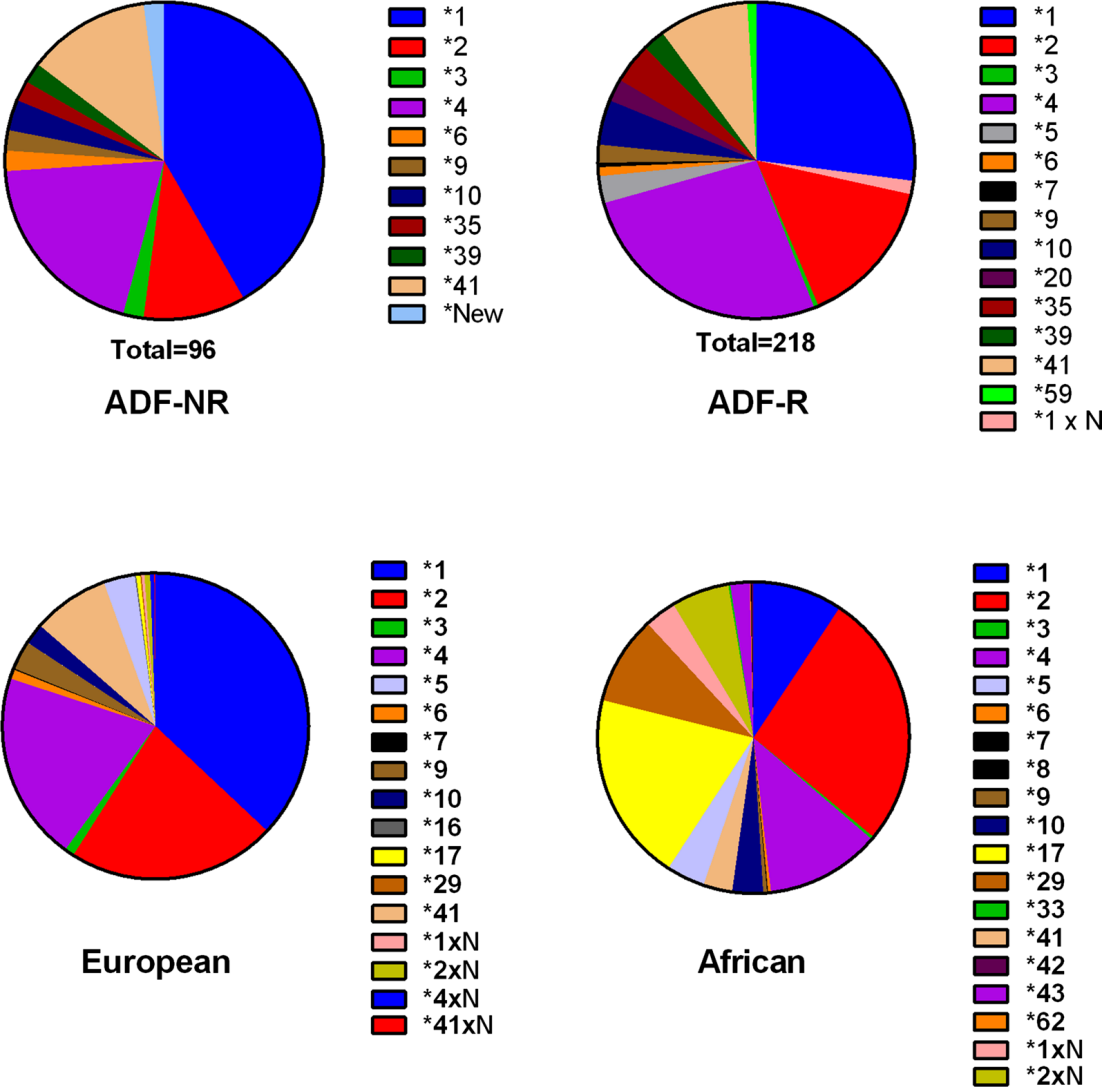
CYP2D6 allele type and their frequency in ADF personnel (ADF-NR and ADF-R) and reference populations (European and African population)

A total of 16 CYP2D6 alleles were observed amongst the 157 ADF samples examined: 10 alleles were observed in the ADF-NR group and 15 in the ADF-R group. Allele type and frequency is shown in Fig. 1 and Table 2. Major alleles that each accounted for > 5% of the ADF-NR group were *1 (41.7%), *4 (19.8%), *41 (12.5%), and *2 (10.4%). These same major alleles were present in the ADF-R group: *1 (27.1%), *4 (26.6%), *2 (15.1) and *41 (9.2%). A single sample from the ADF-NR group had a deletion spaning from 1023 to 1863 bp in the sequence, for which allelic type has not previously been defined in the existing database and was classified as a new allelic type.

**Table 2:** Functionality of CYP2D6 alleles observed in ADF samples

Functionality	Allele	ADF-NR		ADF-R		European [21]
		No.	%	No.	%	%
Increased function (IF)	*1 × N	0	0.0	3	1.4	0.3
Fully function (FF)	*1	*40*	*41.7*	*59*	*27.1*	*37.0*
	*2	*10*	*10.4*	*33*	*15.1*	*22.0*
	*35	2	2.1	9	4.1	0.0
	*39	2	2.1	5	2.3	0.0
Reduced function (RF)	*9	2	2.1	4	1.8	3.0
	*10	3	3.1	10	4.6	2.2
	*41	*12*	*12.5*	*20*	*9.2*	*8.1*
	*59	0	0.0	2	0.9	0.0
Non-function (NF)	*3	2	2.1	1	0.5	1.0
	*4	*19*	*19.8*	*58*	*26.6*	*20.0*
	*5	0	0.0	6	2.8	3.3
	*6	2	2.1	2	0.9	1.0
	*7	0	0.0	1	0.5	0.1
	*20	0	0.0	5	2.3	0.0
Unknown function	*New type	2	2.1	0	0.0	0.0
Total	16	96		218		

Major alleles are shown in italics. Minor alleles in the European population that were not detected in the ADF groups are not shown in the table

Comparisons of allele frequency for major alleles (> 5%) between the two ADF groups (NR and R) and an European population [21] revealed that allelic profile of the ADF-NR group was not statistically different to the European population (p = 0.054), while the ADF-R group had a significant higher proportion of *4 and lower proportions of *1 and *2 than the European population (p < 0.001, Fig. 1). CYP2D6 allele frequency of an African population is included in Fig. 1 for illustration of a difference in allele frequency between populations. Both ADF groups differed significantly in allele frequency from the African population (p < 0.001). There was a significantly higher proportion of *4 and lower proportion of *1 in the ADF-R group compared to the ADF-NR group (p < 0.001, Fig. 1).

After translating the observed CYP2D6 alleles to functionality (Table 2), there was a significant difference in the ADF-R group compared to both the European (p < 0.007) and the ADF-NR (p = 0.008) groups with increased proportion of NF and decreased proportion of FF/IF alleles in the ADF-R group. There was no significant difference in proportion of functionality classification of the CYP2D6 alleles observed in the ADF-NR group compared to the European population (p = 0.46).

### CYP2D6 allele diplotypes

Homozygous CYP2D6 diplotypes were observed in 33/48 (68.8%) and 39/109 (35.8%) of the ADF-NR and ADF-R groups, respectively. When functionality classification of the diplotypes were analysed, there was a significant difference between the ADF-NR and ADF-R groups with the ADF-R having a higher proportion of FF/NF and RF/NF (p < 0.001) (Table 3). The proportion of diplotypes with at least one IF or FF allele was 68.8% (33/48) and 68.8% (75/109) in the ADF-NR and ADF-R group, respectively. Importantly, individuals having NF/NF diplotypes accounted for approximately 20% in each of the ADF groups. 6.4% of individuals in the ADF-R group had a RF/NF diplotype.

**Table 3:** Number and proportion of CYP2D6 functionality diplotypes in ADF-NR and ADF-R group

Group	No	Allele functionality combinations								
		IF/IF	IF/NF	FF/FF	FF/RF	FF/NF	RF/RF	NF/NF	RF/NF	New/new
ADF-NR	48	0	0	19 (39.6%)	9 (18.8%)	*5 (10.4%)*	4 (8.3%)	10 (20.8%)	*0 (0.0%)*	1 (2.1%)
ADF-R	109	1 (0.9%)	1 (0.9%)	32 (29.4%)	17 (15.6%)	*24 (22.0%)*	6 (5.5%)	21 (19.3%)	*7 (6.4%)*	0 (0.0%)
Total	157			54	26	*28*	11	31	*6*	1

Diplotypes with significant difference between ADF-NR and ADF-R is indicated in italics

### Predicted CYP2D6 phenotypes and activity score (AS)

**Fig. 2:**
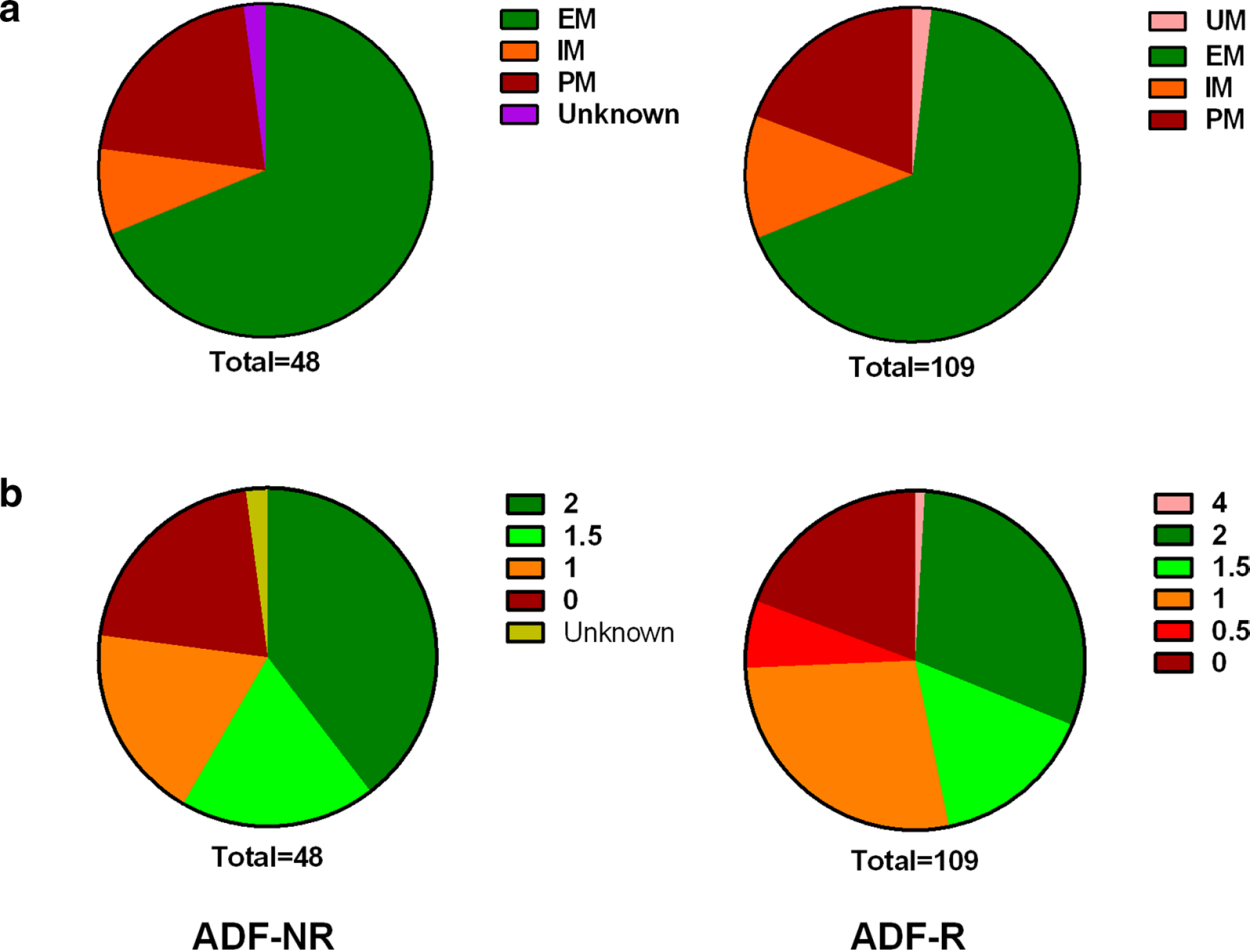
Distribution of predicted CYP2D6 phenotypes (**a**) and activity scores (**b**) in ADF- NR and ADF-R groups

The distribution of predicted CYP2D6 phenotypes between the ADF-NR and ADF-R groups is shown in Fig. 2a. There was no significant difference between the two groups (p = 0.39). A generalized linear model revealed that CYP2D6 phenotype does not significantly influence whether relapse occurred.

Figure 2b shows the distribution of predicted CYP2D6 AS for each ADF group. Although the proportion of samples with AS ≥ 2 was larger in the ADF-NR group than in the ADF-R group (39.6% vs. 31.2%), the proportion with AS = 0 was similar between the two groups (20.8% vs. 19.3% for ADF-NR and ADF-R, respectively). When AS was grouped as AS < 1.5 and AS ≥ 1.5 there was no significant association with ADF groups (p = 0.165). The ROC analysis found that the AS score was not able to significantly discriminate between the two ADF groups (p = 0.30).

### CYP2D6 profile in the ADF-R group with single and multiple relapses

**Fig. 3:**
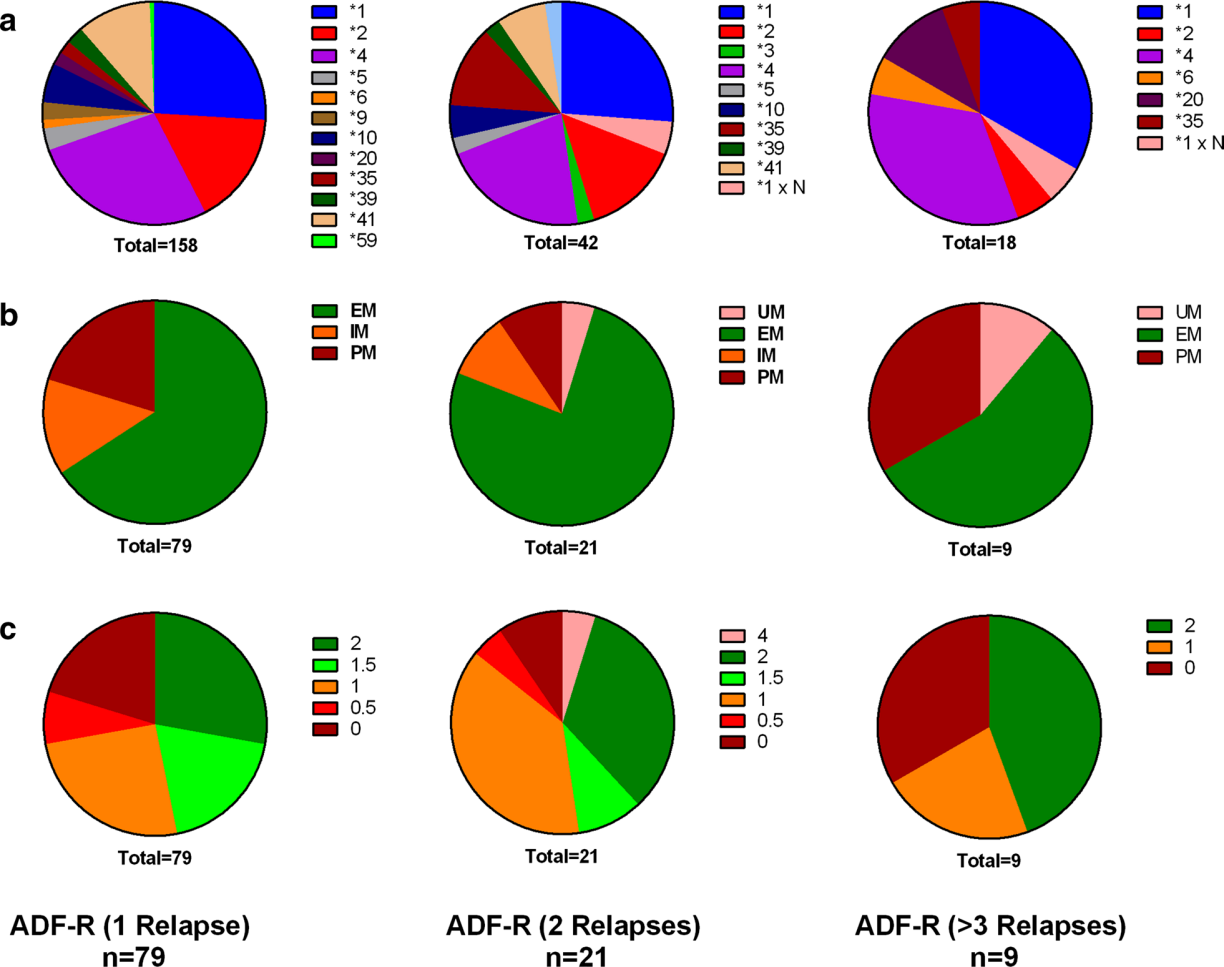
Comparison of CYP2D6 profile within the ADF-R group: **a** allele frequency; **b** predicted phenotype; **c** activity score

Within the ADF-R group, CYP2D6 allele profile was similar between personnel who experienced 1, 2 and 3+ relapses (p = 0.71, Fig. 3a). There was no significant difference in the phenotypes between those who experienced one relapse compared to two or more relapses (p = 0.477, Fig. 3b), and no significant difference in the AS (p = 0.40, Fig. 3c).

## Discussion

Malaria, especially relapsing vivax malaria remains a serious problem for people visiting malarious areas, particularly military populations including the ADF. PART is commonly used as an intervention measure to reduce the incidence of vivax relapses after leaving endemic countries and after relapse. The recommended dose of primaquine used in PART for the ADF was 22.5 mg daily for 14 days (before April 2000) and 30 mg daily for 14 days after April 2000 [15]. However, despite using PART relapsing vivax malaria in returning personnel is common. Within ADF personnel the median period between departing the endemic countries (PNG, East Timor and Solomon Islands) and having a primary presentation of *P. vivax* malaria was 86 days (range 1–505 days) [15]. Almost all cases were considered relapses resulted from hypnozoite activation since most personnel had their primary infection 3 weeks and after their return without further visits to endemic areas.

The aim of this study was to determine the CYP2D6 profile in a subset of ADF personnel deployed to PNG and East Timor and to explore whether their CYP2D6 activity can explain PART failure causing relapsing vivax malaria. Serum and blood samples from personnel deployed to PNG and East Timor were pooled for analysis for the following reasons: (1) both deployments took place within a short time frame (between 1999 and 2000) and a major change in ethnic composition of the troops during this period was unlikely; (2) all personnel received PART; (3) the malaria attack rate for ADF personnel deployed to these two countries were estimated to be similar at 1.1% (52/4776) in PNG and 0.9% (369/40571) in East Timor [15]; and (4) there were insufficient samples from each location to allow for statistically valid country-specific comparisons.

CYP2D6 alleles were determined by DNA sequencing and by PCR detection for copy number variations. The allele frequency determined for the ADF-NR group matched that of the European population, with the same major alleles. While the same four major alleles were also dominant in the ADF-R group, there was a significant increase in the proportion of non-function alleles (*4) and a decrease in proportion of functional alleles (*1), compared to the European population and the ADF-NR group. However, the difference disappeared when allelic types were interpreted into phenotype and ASs. The proportion of diplotypes with at least one functional allele was identical between the two ADF groups at 68.8%. This provides an explanation for why difference in allelic frequency was not translated into difference in phenotype frequency.

Comparable phenotype and ASs were observed between the ADF-R and ADF-NR groups suggesting that CYP2D6 was not the determining factor of whether a relapse occurred or not after PART. Other factors could contribute to relapsing vivax malaria in this cohort including poor adherence to the 14 day PART regimens and tolerance of *P. vivax* to primaquine. For the latter, *P. vivax* strains in Oceania are considered tolerant to primaquine [13]. The majority of ADF personnel returned before April 2000 and were prescribed primaquine at 22.5 mg daily for 14 days, which is equivalent to 0.32 mg/kg based on a body weight for a 70 kg individual. Although not weight adjusted this dose is within the WHO recommended dose range for primaquine (0.25–0.5 mg/kg daily for 14 days). However, the dose may not be sufficient to kill all hypnozoites in the liver. The current recommended primaquine dose for the ADF is 30 mg daily for 14 days.

A limitation of the study was that administration of primaquine was not directly observed as the PART regime is routinely prescribed for returning ADF personnel. Findings from field trials have shown significantly higher recurrence of *P. vivax* infections in unobserved primaquine treatment arms compared to directly observed primaquine treatment [9, 11].

In this study, the investigation into the CYP2D6 status of the ADF personnel in the development of vivax relapses is further complicated because not all personnel in the deployment would be expected to have been bitten by *P. vivax*-infected mosquitoes. While over 400 relapsing *P. vivax* infections reported in the returning personnel suggesting a moderate exposure to *P. vivax* infections [15], there is no guarantee that every individual had exposures during deployment and currently there is no validated marker to quantify exposure. If an individual has not been exposed to *P. vivax* then PART failure would not occur, irrespective of their CYP2D6 status. Therefore, the definitive analysis of the role of CYP2D6 status in the success/failure of PART requires that all individuals are at least exposed to vivax malaria. However, it is not possible to conduct such a study since a key objective during deployment (and travel in general) is to prevent exposure to infected mosquitoes through the use of insecticides, bed nets, repellents and insecticide-impregnated clothing. Future prospective studies should minimize the effect of these confounders.

In order to reduce the potential confounder effect of exposure to infection and adherence to PART, CYP2D6 profile within the ADF-R group was compared between sub-groups who experienced 1, 2 or more relapses. These personnel have all suffered from at least one relapse of the debilitating *P. vivax* infection after their return to Australia and therefore had good incentives to avoid experiencing further relapses, and thus are more likely to adhere to the full course of PART. However, no correlation between CYP2D6 alleles and additional relapses were obtained although there were trends suggesting an association between low AS (0–1) and experiencing multiple relapses. The results indicate that primaquine tolerance may be the major contributing factor to relapses and the higher dose regimen of primaquine (0.5 mg/kg per day for 14 days) should be used for PART.

Although the outcome of this study suggests that the CYP2D6 status in ADF personnel was not the determining factor for relapsing vivax malaria, it does not rule out that CYP2D6 status will affect efficacy of PART in future military operations, especially in individuals having a CYP2D6 diplotype with both non-function alleles. This cohort made up 20% of the current study. Importantly, the study highlights the importance of ensuring adequate primaquine dosing and adherence to PART to reduce the risk of relapsing *P. vivax*. This applies to both military and civilian populations.

## Conclusions

CYP2D6 status was not the dominant factor for PART failure causing relapsing vivax malaria in a cohort of ADF personnel returning from deployment in PNG and East Timor between 1999 and 2001. Other factors such as adherence to PART and primaquine tolerate *P. vivax* are likely contributors to relapses. The results highlight the importance of ensuring adequate primaquine dosing and adherence for preventing relapsing vivax malaria.

## Abbreviations

CYP2D6: cytochrome P450 2D6
PART: primaquine anti-relapse therapies
ADF: Australian Defence Force
ADFMIDI: Australian Defence Force Malaria and Infectious Disease Institute
PNG: Papua New Guinea
ADF-NR: ADF no *P. vivax* relapse
ADF-R: ADF had *P. vivax* relapse
RDTs: rapid diagnostic tests
G6PD: glucose-6-phosphate dehydrogenase
UM: ultrarapid metabolizer
EM: extensive metabolizer
IM: intermediate metabolizer
PM: poor metabolizer
IF allele: increased funtion allele
FF allele: fully function allele
RF allele: reduced function allele
NF allele: non-function allele
AS: activity score
ROC analysis: receiver operating characteristic curve analysis
